# Intelligent Beam-Hopping-Based Grant-Free Random Access in Secure IoT-Oriented Satellite Networks

**DOI:** 10.3390/s25010199

**Published:** 2025-01-01

**Authors:** Zhongliang Deng, Yicheng Liao

**Affiliations:** School of Electronic Engineering, Beijing University of Posts and Telecommunications, Beijing 100876, China; dengzhl@bupt.edu.cn

**Keywords:** LEO satellite, grant-free random access, Internet of Things (IoT), beam-hopping

## Abstract

This research presents an intelligent beam-hopping-based grant-free random access (GFRA) architecture designed for secure Internet of Things (IoT) communications in Low Earth Orbit (LEO) satellite networks. In light of the difficulties associated with facilitating extensive device connectivity while ensuring low latency and high reliability, we present a beam-hopping GFRA (BH-GFRA) scheme that enhances access efficiency and reduces resource collisions. Three distinct resource-hopping schemes, random hopping, group hopping, and orthogonal group hopping, are examined and utilized within the framework. This technique utilizes orthogonal resource allocation algorithms to facilitate efficient resource sharing, effectively tackling the irregular and dynamic traffic. Also, a kind of activity mechanism is proposed based on the constraints of the spatio-temporal distribution of devices. We assess the system’s performance through a thorough mathematical analysis. Furthermore, we ascertain the access delay and success rate to evaluate its capability to serve a substantial number of IoT devices under satellite–terrestrial delay and interference of massive connections. The suggested method demonstrably improves connection, stability, and access efficiency in 6G IoT satellite networks, meeting the rigorous demands of next-generation IoT applications.

## 1. Introduction

As satellite communication technology continues to advance, it is now capable of serving users in various environments such as air, sky, earth, and sea. This has led to an increased demand for satellite communication in different scenarios, with users expecting higher communication quality. Currently, the international organization responsible for standardizing communication and prominent research institutions has prioritized global seamless high-speed coverage as a key area of study for the sixth-generation mobile communication technology (6G) [1,2]. This research focuses on addressing high-speed communication challenges in various scenarios such as satellite connections, seismic communications, military communication, ocean communication, desert environments, and other situations where there are limitations on the number of users. The advancement of satellite communications and the secure Internet of Things (IoT) has positioned satellite IoT networks as a critical application scenario in both the present and future [3]. However, they face challenges in providing the same level of network service performance as terrestrial Fifth-Generation Mobile Communication Technology (5G) in special scenarios [4,5,6]. These challenges include dealing with significant transmission delays, limited communication resources, and an uneven distribution of covered users [7]. Given the current performance of network services, it is imperative to investigate the communication issues in the satellite end scene to achieve worldwide uninterrupted, fast, intelligent and highly dependable communication [8].

In considering the terrestrial mMTC scenario, it becomes evident that traditional grant-based random access is afflicted by two significant shortcomings [9,10,11]. Firstly, the probability of failure for grant-based random access at a large scale will be high, resulting in a significant delay in access [12]. Secondly, large-scale grant-based random access is characterized by a significant signal overhead, which ultimately reduces the efficiency with which wireless resources are utilized [13]. In this context, the design of a large-scale random access system with low latency and high reliability is necessary to accommodate the specific characteristics of satellite Internet of Things. To address the challenges posed by large-scale random access, the academic community has put forth a grant-free random access (GFRA) technology [14]. The fundamental concept of grant-free random access is that, following the transmission of the preamble by an active device, the data signal can be sent directly without awaiting authorization from the satellite. This approach markedly reduces the access delay and signaling overhead associated with large-scale access. It is therefore widely considered that large-scale unlicensed random access may represent a potential technology for 6G wireless networks [15]. The sporadic nature of IoT services means that the satellite is unable to determine which devices have sent data [13]. However, the key to large-scale unlicensed random access is to detect activated devices based on the received preamble.

In traditional multi-beam communication systems, each beam is fixedly allocated a certain amount of power. This results in a waste of power due to the uneven distribution of ground users. The amplifier aggregates the total power and facilitates the flexible transfer of power distribution. The phased array antenna is capable of modifying the antenna pattern through the manipulation of the phase of the feed, thereby enabling the flexible configuration of the coverage area size, coverage area position, and number of beams. This allows for the achievement of beam scanning and the attainment of comprehensive on-demand coverage of terrestrial beam positions [16]. Hence, it is imperative to address the issue of user communication interference in satellite scenarios in order to establish effective communication and maximize efficiency for achieving low-latency and dependable access. Satellite communication involves a significant quantity of terminal equipment, several types of equipment, and a high likelihood of message collision. As the number of users increases, the likelihood of user collision also significantly increases, leading to a decline in all other evaluation metrics [17]. Hence, it is imperative to address the issue of user communication interference in satellite scenarios in order to establish effective communication and maximize efficiency for achieving low-latency and dependable access. This study assumes that the altitude of Low Earth Orbit (LEO) is 600 km, leading to a two-way propagation delay of four milliseconds and a transmission delay of one millisecond. This is attributable to the prevalence of short packet transmission in massive Machine-Type Communications (mMTC) [18,19]. Considering the duration required for propagation, the processing time is relatively brief. An access slot is established to have a duration of six milliseconds [20]. The contention timer in the satellite connection can be configured to fifty milliseconds [21], allowing the transmission frame in this S-IoT system to accommodate eight access slots.

In general, a large number of devices tend to transmit packets and signals to LEO satellites in a sporadic or periodic manner. In order to address the limitations of authorization-based random access, ref. [22] proposes an authorization-free random access protocol. In large-scale authorization-free random access, the base station is not required to perform dynamic scheduling authorization on an ongoing basis. Instead, a just-in-time transmission method is employed, whereby data are transmitted by a device as soon as it becomes available. It is possible that the traditional grant-based access scheme may prove inadequate for supporting the substantial number of connections anticipated in 5G networks. In light of the considerable projected increase in the number of UEs in future satellite IoT scenarios for mMTC applications [23], dedicated preamble allocations in traditional grant-based schemes are likely to be impractical. In the context of the GFRA protocol, two distinct types of resource management schemes have been identified: the random selection type and the pre-configuration type. In a GFRA-based random selection system, each activated user equipment (UE) randomly selects a preamble sequence from a predefined set and transmits it to the satellite along with the data payload. This can serve to mitigate the effects of system overload and preamble conflicts [24]. In the context of random-access GFRA, a more practical approach is proposed in [25], where the authors designed a novel random access scheme based on its fixed TA information. As the quantity of accommodated devices rises, the distribution of time–frequency resources may become limited. Thus, the satellite must utilize grant-free random access to markedly diminish access latency and signaling overhead, and this does not require higher computational requirements and complexity. Table 1 discusses and compares the advantages of grant based access and grant free access.

Inspired by the above discussions and comparison, this paper puts forward a novel grant-free random access protocol, based on beam-hopping (BH-GFRA), which is designed to allocate radio resources in accordance with the beam-hopping pattern. The BH-GFRA protocol is specifically designed to address the challenges of IoT-oriented satellite networks by enhancing access capacity, reducing latency, and ensuring high reliability. It achieves high access capacity by optimizing resource allocation and scheduling, allowing multiple IoT devices to connect and transmit data concurrently. To meet the low latency requirement, BH-GFRA employs a time-slot-based scheduling strategy, minimizing waiting times for devices, while prioritizing latency-sensitive data for faster transmission. Furthermore, high reliability is ensured through advanced channel estimation, error detection, and correction techniques, which mitigate the impact of signal attenuation and interference, ensuring robust data transmission even in challenging network conditions. In the proposed BH-GFRA, satellite and IoT devices access the network according to a time-sequenced beam-hopping pattern. This is achieved by transmitting their pre-allocated preamble to announce their activities. We also note that different types of terminals in the IoT initiate access at different times, so we reduce the impact of resource collisions between terminals by controlling the admission of different types of users in the time slots of the frequency-hopping pattern based on the constraints of the spatial and temporal distribution of users and satellites. The satellite transmits a data packet in the same slot as the beam-hopping pattern that is associated with the preamble. The probability of a collision in the frequency-hopping pattern is subjected to mathematical analysis, as is the capacity of the BH-GFRA protocol, to accommodate a specified maximum data transmission delay requirement.

The remainder of the work is organized as follows: Section 2 presents the system model of this thesis and the procedure of the proposed beam-hopping-based grant-free random access; in Section 3, the resource-hopping mechanism is analyzed; Section 4 describes the performance metrics; Section 5 offers some simulation of this algorithm; and finally, Section 6 concludes this work.

## 2. System Model and Procedure of BH-GFRA

### 2.1. System Model

**Figure 1:**
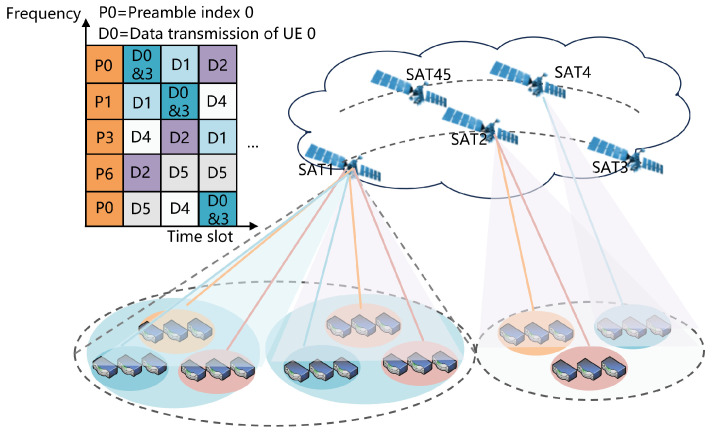
Scenario of BH-GFRA.

In this paper, we consider the forward link transmission of the multi-beam satellite system consisting of multiple NGSO satellites, as depicted in Figure 1. There are *N* satellites serving the ground area, which contains a total of *M* cells. The sets of satellites and cells are denoted by N={n∣n=1,2,…,N} and M={m∣m=1,2,…,M}. In particular, the set of cells covered by the *n*-th satellite is represented by $M_{n}$, and we assume that each satellite can cover *J* cells (i.e., $\left|M_{n}\right|=J,\forall n\in$N). The cell index set under the coverage of one satellite is denoted as J={j∣j=1,2,…,J}. Due to the multiple coverage characteristics of the NGSO satellite communication system, one cell may be covered by multiple satellites, such that $M={⋃}_{n\in N}M_{n}$ and $M_{i}\cap M_{j}\neq \emptyset ,\forall i,j\in N$. In this system, each satellite can simultaneously generate beams to serve distinct IoT scenarios using a time division multiple-access approach, with each beam covering a single scenario. This manuscript concentrates on time-slot-driven scheduling for terrestrial IoT scenarios, wherein the beams of satellites must be scheduled for each time slot, and the time–frequency resource grid is allotted through a beam-hopping technique. The collection of served cells during each time slot is referred to as the BH pattern.

We focus on the GFRA random access protocol for IoT-based satellite access network systems, where a large number of IoT devices send data packets to the satellite intermittently, but can only communicate when the beam slot is lit by the beam. In our system, the sequence of lit slots is structured as a hopping beam. Various time slots are allocated for distinct satellite services; therefore, it is necessary to create a frequency–time resource grid and designate the index of each slot together with the corresponding sent information, referred to as the BH-GFRA block. Initially, we posit that the GFRA blocks has G=R·L resources in the header and R·R resources in the data segment. Each resource block is tasked with occupying a time slot, the length of which is dictated by the subcarrier interval designated for transmission. This interval is represented by the symbol $T_{slot}$. The load factor, represented by *L*, clearly shows the traffic situation of device in J cell, with each corresponding to the $i_{th}$ GFRA group, under the assumption that Internet of Things devices can attempt to execute GFRA within J cell. Each device can transmit data at the $i_{th}$ random access occasion (RO) during the $I\cdot T_{slot}$ time interval. Subsequently, we will introduce the proposed BH-GFRA approach within a singular GFRA block, ensuring the prompt elucidation of our explanation.

If at least one data packet is chosen for transmission, all active devices within the $i_{th}$ GFRA group are operational. In the absence of this condition, the gadget remains inactive. Consequently, the quantity of active devices within each GFRA block varies in line with the variable *v*. This study’s findings indicate that device activation probability varies due to the sporadic need for data transfer from devices situated in distinct places. The activation probability *v* influences the quantity of active devices within a particular GFRA group. This is approximately characterized as *v* multiplied by *R* and *L*. In each GFRA group, let $preamble=\{{preamble}_{1},{preamble}_{2},\ldots ,{preamble}_{\tau }\}$ denote a designated preamble set, where τ signifies the number of uniformly-length preamble sequences available. Each active device that wants to transmit uplink data sends a predetermined preamble as its signature in the preamble section to allow the satellite to identify which devices have attempted GFMA as compared to the random preamble selection. We can utilize the Zadoff-Chu (ZC) sequence as the preamble signal. The satellites then detect ZC sequence-based priors by calculating the correlation value and investigating whether the correlation value exceeds the detection threshold for each prior detection region. After identifying the active devices, the satellite obtains an active list of all devices on a specific GFMA block.

Moreover, we analyze the time-domain composition of the PRACH resource form mentioned above. We conclude that there are ${pr}_{slot}$ PRACH time slots in a cycle $T_{PRACH}$, and each time slot has ${Num}_{Ro}$ access occasions. Therefore, the entire PRACH time slot resource can be represented by a function matrix:

(1) $$\xi \left(T_{PRACH}\right)=\left[\begin{array}{cccc} {\mathrm{RO}}_{11} & {\mathrm{RO}}_{12} & \cdots & {\mathrm{RO}}_{1j}\\ {\mathrm{RO}}_{21} & {\mathrm{RO}}_{22} & \cdots & {\mathrm{RO}}_{2j}\\ \vdots & \vdots & \ddots & \vdots \\ {\mathrm{RO}}_{i1} & {\mathrm{RO}}_{i2} & \cdots & {\mathrm{RO}}_{\mathrm{ij}} \end{array}\right]$$

The formula $\xi (T_{PRACH})$ represents the distribution of RO resources in a PRACH cycle, and ${RO}_{ij}$ is denoted as the *j*th RO resource in the *i*th PRACH time slot, where i=1,2,3…, ${pr}_{slot}$ and $j=1,2,3,\ldots ,{Num}_{RO}$.

Based on the PRACH resource matrix, this paper establishes the resource consumption and model of the satellite and the space–time distribution constraint model of the device side to achieve access resource demand [41]. In this scenario, beam-hopping satellite coverage is used, and devices in a specific region are configured to be $U=\{u_{1},u_{2},\ldots ,u_{n}\}$, the satellite coverage type in this scenario is beam-hopping coverage, and the devices are classified as $U_{sens}=\left\{u_{ts1},u_{ts2},\ldots ,u_{tsi}\right\}$ time-sensitive devices and $U_{sparse}=\left\{u_{st1},u_{st2},\ldots ,u_{stj}\right\}$. $U_{num}=\left\{U_{sens}+U_{sparse}\right\}$ is the total number of connected devices, where $U_{sens}$ represents the number of devices with time constraints and $U_{sparse}$ represents the number of regular devices, which just send some sparse data traffic. By constructing a device distribution constraint model [42], $F\left(S,D,P\right)=F_{S}*F_{D}*F_{P}$ based on the spatial location *S*, device density intensity *D*, and device distribution model *P* [43]. The device distribution location *S* is associated with the global latitude and longitude coordinates (X,Y), and a certain device is set to be $U=\{u_{1},u_{2},...,u_{n}\}$. Then, the location where and when traffic occurs is set as $\left(X_{r},Y_{r}\right)$ and the space range as $L_{s}$. $U_{sparse}\left(L_{s}\right)$ and $U_{sens}\left(L_{s}\right)$ represent the variances in the number of time-sensitive and normal devices in different locations. Along with the time parameter *t* of the number of devices’ time expression for $U_{sparse}\left(L_{s},t\right)$ and $U_{sens}\left(L_{s},t\right)$, different moments of the number of devices will also vary [44]. The probability of an overall device initiating access, which indicates changes in the number of devices gaining access, is the main topic of this work. The probability of access for a time-sensitive device is ${prob}_{sens}$, whereas that of a normal device is ${prob}_{sparse}$. As a result, the number of applicants in a region at a given time during the random access procedure can be written as follows:

(2) $$U_{num}\left(L_{s},t\right)=U_{sparse}\left(L_{s},t\right)*prob_{sparse}+U_{sens}\left(L_{s},t\right)*prob_{sens}$$

### 2.2. Procedure of GFRA

**Figure 2:**
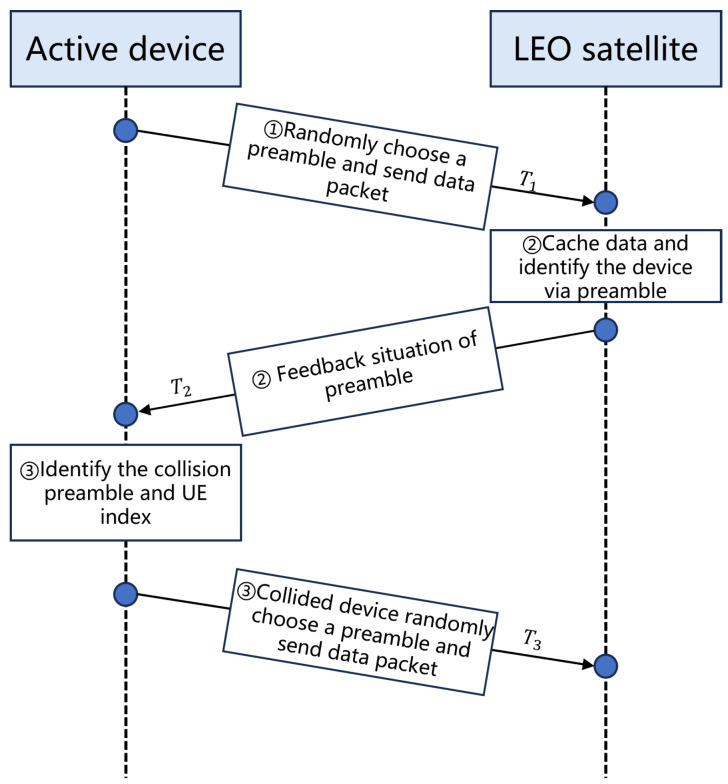
The procedure of the proposed GFRA protocol.

Figure 2 illustrates the procedure of the proposed GFRA protocol. To execute more grant-free random access in an S-IoT network, the GFRA mechanism undergoes the following phases:

Step 0: When the spotbeam of the satellite only lights the beam position, the satellite will send a signal to J cell. Also, 3GPP defines that after receiving the satellite downlink Synchronization Signal Block (SSB), the device starts to select the preamble and sends an access request to the appropriate random access occasion (RO). Here, we assume that the device must start receiving the SSB after transmitting data, and the probability of the device being active defines the process by which the device successfully completes synchronization.

**Step 1**: Each of the $L_{device}$ devices randomly selects a preamble from the assigned preamble P and transmits it within the first slot of the GFRA group.

**Step 2**: The satellite receives the preamble and then allocates the time–frequency resources for data transmission to this device based on the received preamble index and the IoT device identifier of the device. The specific frequency-hopping pattern will be mentioned later. At the beginning of the access slot, the satellite uses the signal power and the first received channel state information (CSI) to identify the state of the preamble sequence. There are three possible preamble sequence occupancy states:

Case 1: If no UE has selected the preamble, the preamble is referred to as an idle preamble.

Case 2: If only one UE has selected the preamble, the preamble is referred to as a single-instance preamble. The satellite detects the single preamble state and assigns the frequency-hopping pattern for data transmission immediately after the current preamble transmission frame ends.

Case 3: If a single preamble is selected by multiple devices, the contending user transmits data according to the resource-hopping pattern specified by the preamble.

**Step 3**: When the beam again covers this beam position, the satellite transmits the preamble usage of the previous access period to all users in the beam position, and each UE can independently detect whether it has a conflict. Each conflicting UE then selects a random preamble sequence from the set of preambles and updates the frequency-hopping mode of the device that selected the previous preamble, and then no feedback will be provided by the satellite until the conclusion of the last access time session.

Finally, the satellite will record the time from the start of access to the correct transmission of data during the first slot scan cycle of the recorder. If the device does not collide when initiating the first access and correctly selects the preamble of the single instance, the access delay is T1; if the device collides when selecting the preamble and successfully transmits data with the satellite during the second scan of the beam for this beam position, the access delay of this device is T1+T2+T3. The number of devices in each beam position is different, and in the following statistics, the average access delay of the users in a selected beam position is selected for the statistics.

## 3. Beam-Hopping-Based Grant-Free Random Access

In this section, we propose an intelligent new access framework for the LEO satellite Internet of Things. In response to the abovementioned discussion, to the best of our knowledge, large-scale IoT access does not use signaling interactions to increase the access success rate, which can cause signaling storms. Furthermore, in the satellite-to-terrestrial scenario, the conflict between agile beam switching frequency, resource scheduling period, and service latency requirements needs to be resolved. Therefore, we designed a direct frequency-hopping sequence according to different preambles in the time slot of the beam-hopping pattern for data transmission. In addition, transmission constraints were imposed by the spatio-temporal distribution of different devices, and the probability of user activation was iteratively generated. It also better classified time-sensitive services and sparse data transmission services, improved the overall access success rate under this framework, and reduced transmission delay.

### 3.1. Resource-Hopping Mechanism

The proposed hopping schemes facilitate the utilization of shared resources by multiple devices in a non-orthogonal manner. In this section, we virtualize the resource grid into a matrix and put forward resource-hopping schemes as matrix transformations. The proposed resource-hopping schemes are as follows: (1) orthogonal frequency-hopping pattern, (2) random hopping pattern, and (3) group-divided hopping pattern. The number of devices in a single GFRA group *G* is assumed in all three schemes. Therefore, the proposed resource *R* hopping schemes are used to reduce resource collisions in IoT devices and increase user capacity in IoT scenarios.

(1) Orthogonal Frequency-Hopping (OFH) Pattern: Orthogonal frequency-hopping (OFH) is a scheme for allocating spectrum resources using orthogonal frequency-hopping sequences, ensuring that each user’s hopping sequences do not overlap in the frequency domain. OFH can significantly reduce interference between users and improve the utilization of spectrum resources. Orthogonal sequences ensure that the probability of frequency collisions between different users is low. However, in IoT, generating massive orthogonal sequences requires more computational resources. In this paper, however, we use an alternating iterative algorithm to reduce the frequency of OFH. It will be mentioned in the following algorithm. The load factor, denoted by the symbol *L*, and the satellite provide a unique means of data transmission. Thus, no resource conflict is present in this scenario. Conversely, when G>R, there may be orthogonal frequency-hopping sequences of size R with ⌈G/R⌉=L. For the $Q^{l}$ OFH matrix, the element at (i,j) is expressed as

(3) $${\left[Q^{l}\right]}_{i,j}=\left(l\times i+j\right)\;\mathrm{mod}\;R$$

where ${\left[\cdot \right]}_{i,j}$ signifies the (i,j) element of the matrix, and mod denotes the modulo operator. An OFH is characterized as a collection of devices that transmit packets over RBs in accordance with a uniform Latin square matrix. In the BH-GFRA system, (3) denotes the index of devices associated with the *l*-th OFH group, allocated to the *i*-th frequency and *j*-th time resource. In actuality, a single resource block may have numerous subcarriers and OFDM symbols, as observed in 3GPP.

**Figure 3:**
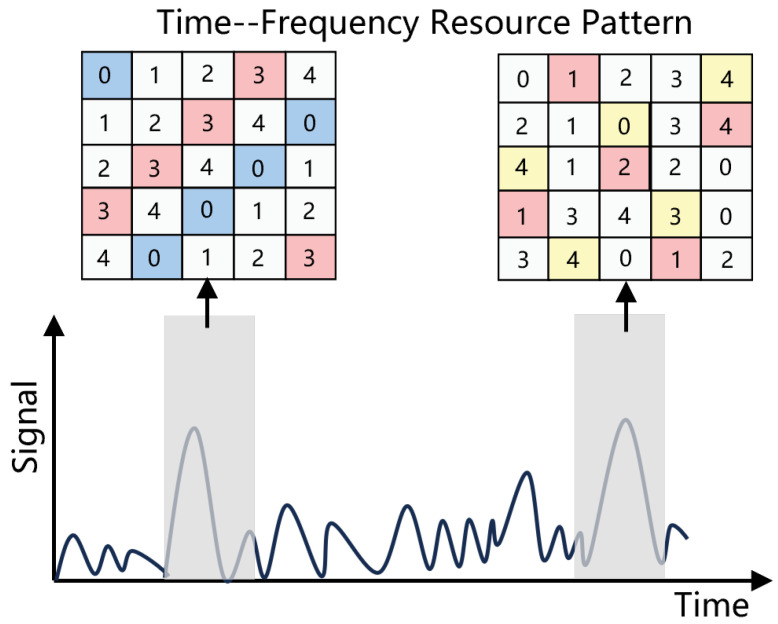
An example of a random hopping pattern-based resource scheme.

Figure 3 illustrates an instance of the collection of OFH square matrices. Each device inside the BH-GFRA group possesses a distinct hopping pattern, hence effectively precluding the occurrence of resource conflicts within the group. Nonetheless, when there are *L* OFH groups, a device may unsuccessfully transmit the data to the satellite because of the interfering devices from distinct OFH groups. The likelihood that a single resource is interfered with by *K* devices, given an activation probability *v*, is expressed as

(4) αgroup(K,v)=L−1KvK(1−v)(L−1−K)

From (4), the probability that ($N_{0},\ldots ,N_{L-1}$) resources are collided by other $L_{device-1}$ devices, respectively, is expressed as

(5) PrN0,…,NL−1∣v=RN0⋯NL−1∏K=0L−1αgroup(K,v)NK

where $N_{0}+\ldots +N_{L-1}=R$. The anticipated quantity of resources impacted by *K* interfering devices for a specified activation probability *v* is alternatively expressed as

(6) $$E\left(N_{K}|v\right)=R\cdot P_{\mathrm{group}}\left(K,v\right)$$

(2) Random Hopping Pattern: The patterns of arbitrary resource-hopping may be ascertained by an identifier. The chance of a target device experiencing a resource collision with active devices, using an activation probability *v* and a quantity of available orthogonal resources *R* during a time slot, is expressed as

(7) βrandom(K,v)=G−1KvRK1−vRG−1−K

This paragraph posits that a target device is functional and utilizes a certain orthogonal resource within a designated time window. Consequently, our focus is solely on the remaining (G−1) devices. The activation is characterized by the binomial distribution G−1gvg(1−v)G−1−g, representing the probability that (G−1−g) devices are active while (G−1−g) devices remain inactive. This likelihood is shown as a percentage. Furthermore, $\left(\begin{array}{c} g\\ K \end{array}\right){\left(1/R\right)}^{K}{\left(1-\left[1/R\right]\right)}^{g-K}$ represents the likelihood that *K* active devices among *g* active devices share the same resource as the target device, while the remaining (g−k) devices do not.

(8) βrandom(K,v)=∑g=KG−1g−1gvg(1−v)G−1−ggK1RK1−1Rg−K=vRK∑g=KG−1G−1ggKv−vRg−K(1−v)G−1−g=g−1KvRK∑g=Kg−1g−1−Kg−Kv−vRg−K(1−v)g−1−g=g−1KvRK1−vRg−1−K

From (8), the probability that $\left({L_{device}}_{0},\ldots ,{L_{device}}_{G-1}\right)$ resources are collided by *G* interfering devices, respectively, is given by

(9) Pr(Ldevice0,…,LdeviceG−1|v)=RLdevice0⋯LdeviceG−1×∏K=0G−1βrandom(K,v)LdeviceK

where ${L_{device}}_{0}+\ldots +{L_{device}}_{G-1}=R$. The expected amount of resources collided by *K* interfering devices for a specified activation probability *v* is also expressed as

(10) $$E\left(L_{deviceK}|v\right)=R\cdot \beta_{\mathrm{random}}\left(K,v\right)$$

(3) Group-Divided Hopping (GDH) Pattern: Analogous to orthogonal hopping, once the device accidentally chooses the single preamble, the hopping scheme that serves as data transmission is the same, hence preventing any resource clashes. Nevertheless, as each group employs the same method that filled the time–frequency slot, and as there is a likelihood that an individual device engages in a BH-GFRA with interfering devices from a different group using the activation probability *v* as an example, there is no difference in the resource-hopping matrix between any of the resource groups. Due to the fact that all devices in each group are operational, additional devices that select the same preamble are subject to collisions with two devices that disrupt the data transmission throughout the process. Also, the other group will not experience the same collisions due to the GDH hopping scheme. A complete packet containing *R* resources is subjected to interference from *K* devices from various groups with a probability of $\delta_{\mathrm{group}}\left(K,v\right)$, which is described as

(11) δgroup(K,v)=L−1KvK(1−v)(L−1−K)

### 3.2. Optimization of Activation Factors by Exploiting Space-Time Distribution Constraint

In the abovementioned system model, the construction and constraints analyses show that the random access procedure is affected by both the number of users initiating access and the effects of retransmission on users in the cycle after a collision. During the access process, the user’s probability of avoiding retreat is denoted as $\rho_{t}$. After a device collision occurs, the retransmission timing is determined using a discrete Gaussian distribution. Hence, the total number of users involved in the access process can be represented as

(12) $$\begin{array}{cc} U_{u}\left(L_{s},t\right)= & U_{num}\left(L_{s},t\right)+U_{num}\left(L_{s},t\right)*\rho_{t-1}-\\ \; & U_{num}\left(L_{s},t\right)*\rho_{t+1} \end{array}$$

where the user’s avoidance probability is set to be ρ during the access process, and after the user collision, the user is obtaining retransmission timing in the form of a discrete Gaussian distribution.

The user’s retransmission timing can be determined by considering the system occupancy. This paper calculates the probability of retransmission in a collision by determining the number of retransmissions. The user who needs to access it again can choose this type of preamble, leading to a more balanced distribution of resources and an overall improvement in performance.

The disparate propagation delays between users and the satellite result in varying times for users to receive access message feedback. In a given moment, user feedback may be received simultaneously by multiple ROs that collided in the previous access cycle. Accordingly, this paper assumes that the number of colliding users at a given time follows a distribution with density function $\rho_{T}$. This distribution is described by a Gaussian distribution with a mean equal to the average transmission delay, $\mu_{t}$, and a user set variance of $\sigma^{2}$. From the aforementioned grant-free access process, it can be observed that the time window size is represented by $t_{win}$. Consequently, the change in user access following a user collision is given by $U_{u}\left(L_{s},t\right)$. Furthermore, the time range within which the collision user will be obtained is set within the time window of $\left[-t_{\mathrm{win}}/2,t_{\mathrm{win}}/2\right]$.

(13) $$U_{col}\left(L_{s},t\right)=\sum_{i=\mu_{t}-t_{\mathrm{win}/2}}^{\mu_{t}+t_{\mathrm{win}}/2}U_{num}\left(L_{s},t-T-i\right)*\rho_{T-i}$$

where $U_{col}\left(L_{s},t\right)$ is the number of collisions.

(14) $$\begin{array}{cc} \; & U_{u}\left(L_{s},t\right)=\\ \; & U_{\mathrm{num}}\left(L_{s},t\right)+U_{\mathrm{num}}\left(L_{s},t\right)*\rho_{t-1}-U_{\mathrm{num}}\left(L_{s},t\right)*\rho_{t+1}+U_{\mathrm{col}}\left(L_{s},t\right) \end{array}$$

By combining the previously derived collision probability for sensitive users, it can be seen that this formula is

(15) $$\begin{array}{cc} \; & U_{u}\left(L_{s},t\right)=U_{\mathrm{num}}\left(L_{s},t\right)+U_{\mathrm{num}}\left(L_{s},t\right)*\rho_{t-1}-U_{\mathrm{num}}\left(L_{s^{\prime }},t\right)*\rho_{t+1}+\\ \; & \sum_{i=\mu_{t}-t_{\mathrm{win}}/2}^{\mu_{t}+t_{\mathrm{tint}}/2}U_{\mathrm{num}}\left(L_{s},t-T-i\right)*\left(1-\alpha_{\mathrm{sens}}\right)\rho_{T-i} \end{array}$$

where $\rho_{T}$ is described by $f\left(T\right)=\frac{1}{\sqrt{2\pi }}\exp\left(-\frac{{\left(T-\mu \right)}^{2}}{2\sigma^{2}}\right)$. Accordingly, the formula for the change in the total number of users can be derived as follows:

(16) $$\begin{array}{c} U_{u}\left(L_{s},t\right)=U_{\mathrm{num}}\left(L_{s},t\right)+U_{\mathrm{num}}\left(L_{s},t\right)*\rho_{t-1}-U_{num}\left(L_{s},t\right)*\rho_{t+1}+\leftarrow \\ \sum_{i=\mu_{t}-t_{\mathrm{win}}/2}^{\mu_{t}+t_{\mathrm{win}}/2}U_{\mathrm{num}}\left(L_{s},t-T-i\right)*\\ \left(1-\frac{{\mathrm{preamble}}_{\mathrm{sens}}*\left({\mathrm{Psens}}_{\mathrm{success}}+{\mathrm{Psens}}_{\mathrm{colision}}\right)}{U_{\mathrm{sens}}\left(L_{s},T-i\right)*\mathrm{probsens}}\right)\frac{1}{\sqrt{2\pi }\sigma }\exp\left(-\frac{{\left(T-i-\mu \right)}^{2}}{2\sigma^{2}}\right) \end{array}$$

Once the requisite access resources have been obtained, the network will ascertain whether these resources are sufficient to meet the access requirements of all time-sensitive users, taking into account the current number of time-sensitive and sparse traffic users. In the event that the aforementioned conditions are not satisfied, the user will determine whether the access demand of the time-sensitive user is satisfied in the subsequent time slot, according to the time-sensitive user change curve. In the event of satisfaction, the user in question will refrain from accessing the network with some time-sensitive users, in accordance with the constraints outlined. In the event of non-satisfaction, the user will proceed with initiating access within the designated time slot. Based on the probability of time-sensitive $Pro_{sens}$ and collision $Psens_{collision}$ situations, the active rate mentioned above will always change. The specific algorithm is detailed in Algorithm 1.

**Algorithm 1:** Optimization of Activation Factors by Exploiting Space–Time Distribution Constraint

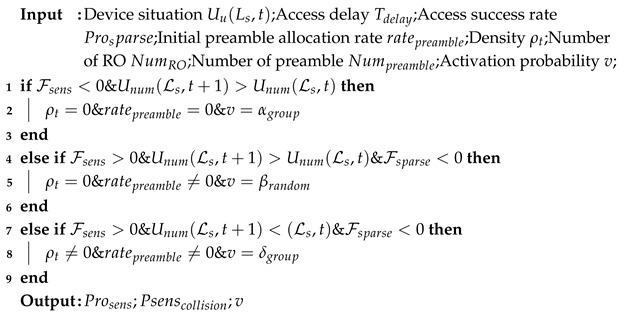



## 4. Performance Metrics of the Proposed GFRA

In this scenario, performance limitations include random access success rate, random access delay, and block error rate. A BH-GFRA is then continuously implemented to maximize user performance. A user collision occurs when the satellite decodes a preamble and receives multiple CSI. Only one device can send successful data to a satellite, while the rest must send access failure information, per the collision resolution rule. Thus, random access collisions are caused by multiple users selecting the same resource.

The user-access delay refers to the time it takes for the user to send the access request message to the gateway station via satellite, following the initiation of the random access process. It is the duration between the user’s action and the gateway station’s response message, which is provided after processing the request. The access initiation moment of user $u_{i}$ is denoted as $t_{u_{i}}^{start}$, and the access completion moment is denoted as $t_{u_{i}}^{end}$. Therefore, the access delay of this user can be calculated as $t_{u_{i}}^{delay}=t_{u_{i}}^{end}-t_{u_{i}}^{start}$. To effectively evaluate the optimization performance of the algorithm, the statistical user-access delay focuses on the average delay experienced by the user $u_{i}^{success}$ who successfully accessed it in an access RO. Users who failed to access it and did not receive the feedback message are not considered when calculating the delay statistics. In other words, the average delay when there are $N^{success}$ users successfully accessing is

(17) $$t_{RO}^{delay}=\frac{\sum_{i=1}^{N^{success}}t_{u_{i}}^{delay}}{N^{success}}$$

The access success rate refers to the probability that a user will successfully access the randomized access system within a given number of retransmissions, denoted as ${Times}_{retr}$, that the user experiences when attempting to initiate access. A user access failure occurs when the user attempts to retransmit the system after surpassing the maximum number of retransmission attempts. In this article’s system, the reason for re-initiating the calculation is that the beam has scanned past this beam position, that is, the number of times the satellite has scanned past the beam position during the time it took to cover this cell. Suppose that the $L_{device}^{\mathrm{GFRA}}$ user initiates a connection but only the $L_{device}^{\mathrm{success}}$ user can successfully connect to it. The connection delay for this user is

(18) $${\mathrm{prob}}_{RO}=\frac{L_{device}^{\mathrm{success}}}{L_{device}^{\mathrm{GFRA}}}$$

The following metrics block error rate (BLER) was utilized as the metric to evaluate and contrast different kinds of frequency-hopping systems with the already employed grant-based RA technique. In the existing contention-based resource allocation system, each device sends data in Msg3 for a four-step access or MsgA for a two-step access. The satellite is not obligated to cache the device data during the first access. Furthermore, during the competition for the preamble in the initial phase of RA, any device can transmit data utilizing identical resources in the subsequent phase; however, the quantity of devices that disrupt the transmission may differ. Furthermore, if the quantity of detected pilot symbols exceeds the number of devices, the Random Access Response (RAR) message required to identify the frequency position for subsequent signaling transmission may not be received. Consequently, data cannot be sent. Therefore, the reasons that the collision will occur are as follows: W1) the target device can receive the RAR, and W2) the RAR cannot correctly transmit to the device, with probabilities P[W1] and P[W2], respectively, applicable. The block error rate (BLER) of the present solution may be calculated using the subsequent formula:

(19) $$P\left[\mathrm{BLER}\right]=P\left[\mathrm{BLER}|W_{1}\right]P\left[W_{1}\right]+P\left[W_{2}\right]$$

## 5. Analysis of Simulation Results

The simulation specifically examines the collision in the random access process and thoroughly examines the impact of different factors on the user collision process. The random access collision simulation is conducted by adjusting the simulation parameters and scenarios, and utilizing data such as user activities during various time periods. The Table 2 explained the simulation parameters. This allows for the analysis and evaluation of access performance metrics such as access delay, access success rate, and BLER. The efficacy of the proposed access framework is validated, as it can effectively guarantee timely user access and enhance overall access performance.

**Figure 4:**
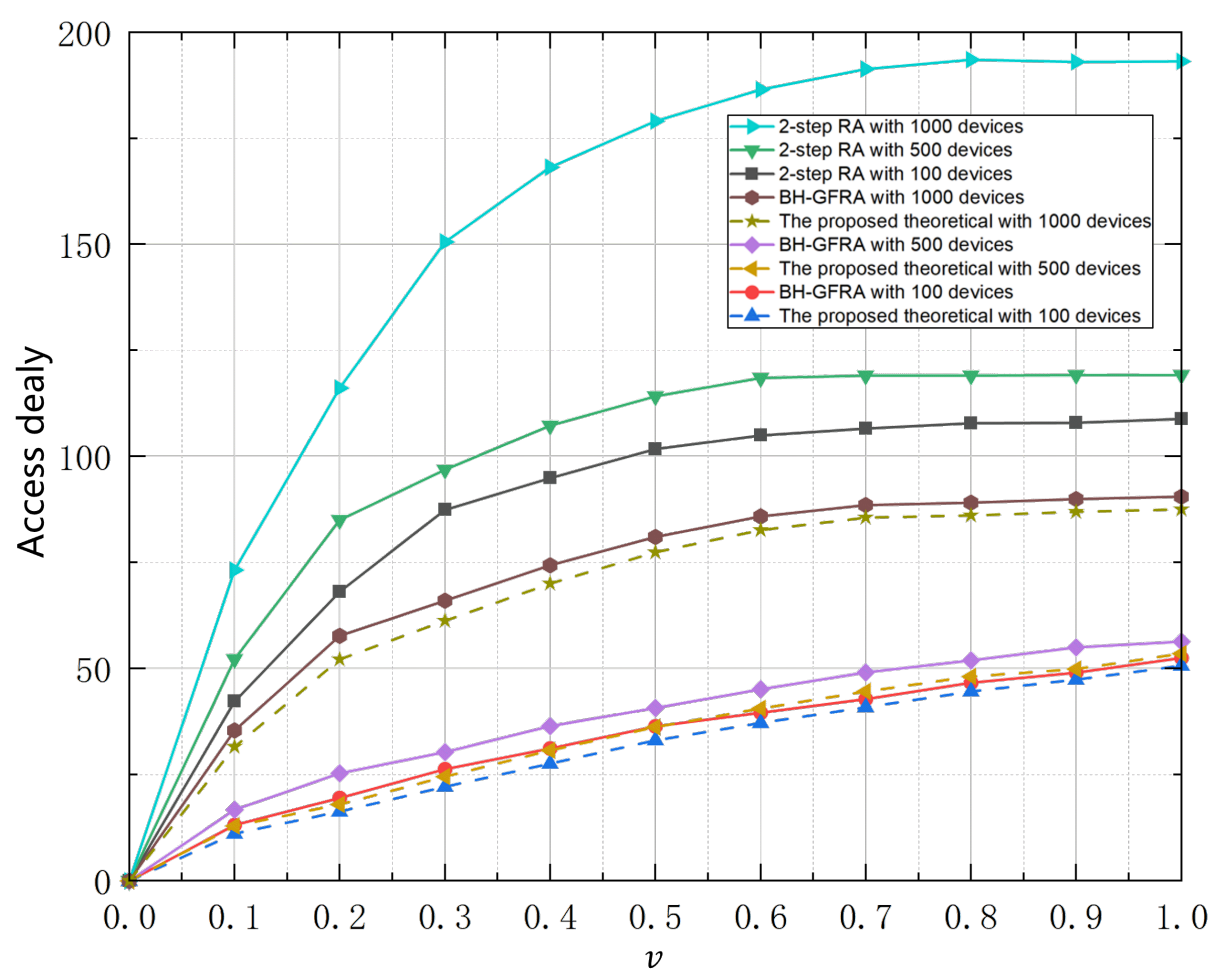
Access delay.

Figure 4 shows a comparison of the access delay of different random access (RA) schemes when the number of devices is 100, 500, and 1000, respectively. The abscissa indicates the access probability, and the ordinate indicates the delay in the system. The exact access delay numerical value is listed in Table 3. The figure compares the theoretical performance of the traditional 2-step RA, BH-GFRA schemes, and the proposed scheme. It can be observed from the figure that, as the access probability increases, the system delay shows a rapid growth trend, especially when the number of devices is large (e.g., 1000 devices), the delay increase is more obvious. This shows that the traditional RA scheme is difficult to effectively reduce the delay under high access load. The theoretical value mentioned is the lowest access delay under different active probabilities *v* obtained through multiple different simulations using three frequency-hopping access methods. The proposed scheme in this paper outperforms 2-step RA in terms of delay under all device numbers, especially in the low-to-medium access probability region (0.1 to 0.5). However, when the access probability continues to increase, the delay of BH-GFRA gradually stabilizes, but it is still higher than the theoretical performance of the proposed scheme. Regardless of the number of devices, the theoretical delay of the proposed scheme is significantly lower than that of the 2-step RA and BH-GFRA schemes. The performance advantage of the proposed scheme is more obvious when the number of devices is small, while the increasing trend of its delay is effectively suppressed when the number of devices is large, showing strong robustness.

**Figure 5:**
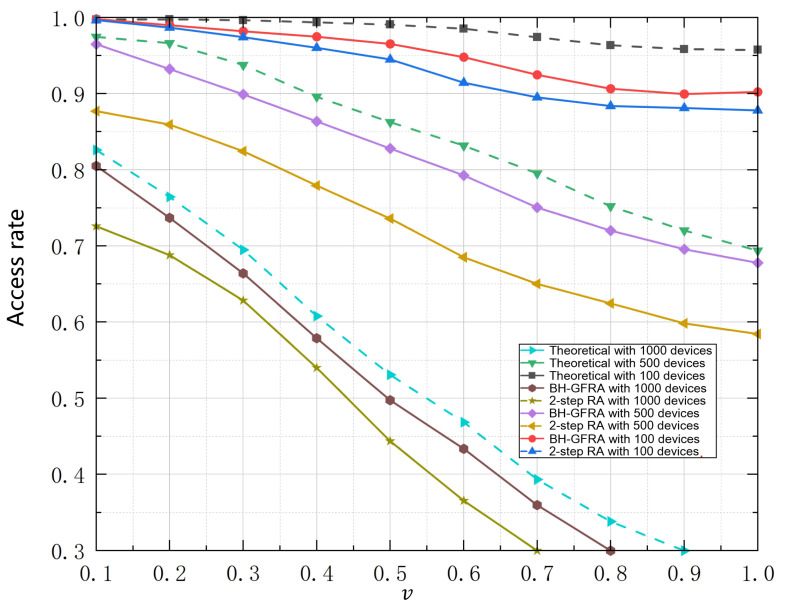
Access success rate.

Figure 5 shows the trend of the access success rate with the normalized load for different numbers of devices and access algorithms. The theoretical analysis in the figure compares two access algorithms (BH-GFRA and 2-step RA). The theoretical access success rate curves show a decreasing trend with device numbers of 1000, 500, and 100, but overall maintain a high success rate, especially in the low-load area (normalized load close to 0.1 to 0.4), where the access success rate is close to 1. As the load increases, the theoretical access success rate gradually decreases, and the success rate increases slightly when the number of devices increases. When the BH-GFRA algorithm is under 1000 devices, its access success rate gradually decreases. When the load is high (e.g., the normalized load is above 0.7), its success rate is significantly lower than the theoretical upper limit. When the number of devices is 500 and 100, the performance of the BH-GFRA algorithm gradually improves, especially in the low-load area, which is close to the theoretical access success rate; but it still shows significant performance degradation in the high-load area. The 2-step RA algorithm shows a similar trend to the BH-GFRA algorithm, but the overall access success rate is slightly higher, especially at 500 devices and 100 devices, and the performance is still better than BH-GFRA when the load is higher. In the low-load area, the access success rate of the 2-step RA algorithm is closer to the theoretical curve, showing better robustness. Also, an increase in device quantity causes the access success rate to decrease significantly with an increase in normalized load, and the difference is more pronounced, especially under high-load conditions. Comparing the three device quantities, the performance of the two access algorithms under 100 devices is closest to the theoretical limit.

**Figure 6:**
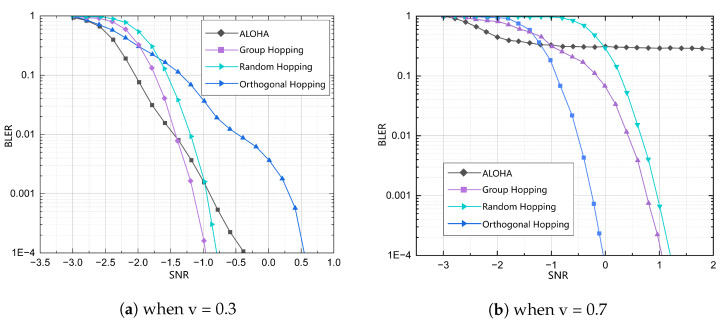
BLER for different values of *v*.

Figure 6 presents a comparison of the block error rate (BLER) for all schemes when the load probability is optimized for different service iterations under time and space constraints. Figure 6a illustrates that the activation probability optimization for the sparse data transmission service is approximately 0.3, which indicates a relatively low device activity level. In this instance, the BLER of the proposed orthogonal frequency-hopping scheme is increased in comparison with the packet, existing grant-based RA, and random frequency-hopping systems, respectively. In contrast, Figure 6b illustrates that for the time-sensitive transmission service, the activation probability is approximately 0.7 after iteration, indicating a relatively high device activity level. In this instance, the BLER of the proposed orthogonal frequency-hopping scheme is superior to that of the random frequency-hopping and packet schemes, exhibiting an improvement of 1.2 and 1 dB, respectively. It is noteworthy that in Figure 6b, the BLER of the existing grant-based RA tends to stabilize when the signal-to-noise ratio reaches a certain threshold. This indicates that improvements in signal quality cannot increase the capacity of the access framework. This also reflects the higher upper limit capacity of the framework proposed in this paper.

## 6. Conclusions

This research primarily examines the BH-GFRA architecture for extensive device access in the satellite Internet of Things (IoT) context. This study examines the modification of user data transmission techniques via the hopping pattern of the hop beam and various resource-hopping methods, aiming to minimize collision risk while adhering to restrictions of access latency and access success rate. Simultaneously, an activation probability optimization strategy is devised to further mitigate secondary user collisions, constrained by spatio-temporal distribution. Simulation findings indicate that BH-GFRA effectively manages extensive access. The investigation of LEO satellite communication networks is in the developmental phase; so, the study presented in this paper may offer valuable theoretical and practical insights for satellite system design.

Despite the improvements in our proposed RA protocols, some problems and challenges in satellite IoT, energy-efficient SDRe mechanisms, and AoI in satellite IoT remain. Heterogeneous IoT devices with different QoS requirements are deployed in different domains experiencing various channel environments. To ensure accurate data detection with minimal AoI, ML-based RA strategies may be a promising solution.

## Figures and Tables

**Table 1:** The advantages versus disadvantages of grant-based access and grant-free access.

Category	Grant-Based Access	Grant-Free Access	Conventional Packet Diversity/Segmentation Approach for Satellite Communication
Advantage	Reduces access collision by controlling the number of IoT devices in each RA slot. Time–frequency resources are divided into different resource blocks (RBs) for different traffic, ensuring QoS requirements such as low E2E latency, high throughput, or low collision [26,27]	Reduces the latency and energy consumption by reducing signaling overhead and collisions; it is suitable for satellite IoT networks and short packet transmissions [28,29,30]	In DVB-RCS2, IoT devices which have low activity, limited energy resources, and short data packets can use the ALOHA protocol and its variants. IoT devices can randomly begin to access an RO (random access occasion) and frequency resources at the same time [31]
Limit	A large number of accesses results in congestion, causing high conflicts, significant signaling overhead, increased resource and energy consumption, and limited uplink resource segmentation	The lack of coordination leads to increased conflicts, challenges in blind data detection, difficulties in identifying activated devices, issues with channel estimation, and synchronization problems in the uplink [32,33]	The increase in the number of IoT devices leads to high collisions, and if a collision occurs and retransmission takes place, the IoT devices may lead to energy leakage, high latency, or packet loss [34]
Related Methods	Adjusts access resources according to load and solves the continuous collision problem after user collision through user avoidance and retransmission	Collision resolution can be achieved with NOMA or massive MIMO, spatial-reuse resource, or dynamic class barring [35,36,37]	Competitive Resolution Diversity Slotted ALOHA and Enhanced Competitive Resolution Diversity Slotted ALOHA are proposed by combining packet diversity transmission and effective interference cancellation techniques [38,39,40]

**Table 2:** Simulation parameters.

Parameter	Value
Height of orbit	600 km
Ka band	20 GHz
Number of device	100, 500, 1000
Time slot duration	10 ms
Simulation duration	10 min
Number of orbits	30
Number of satellites	80
Bandwidth	20 MHz
Number of beam positions/GFRA group	24
Number of preambles	64

**Table 3:** Table of access dealay.

v	Theoretical with 500	BH-GFRA with 100	Theoretical with 100	RA with 500	BH-GFRA with 500	Theoretical with 500	RA with 1000	BH-GFRA with 1000	Theoretical with 1000
0.1	42.2502	13.11317	11.06657	52.22639	16.77225	12.89611	73.20796	35.44779	31.59064
0.2	68.11035	19.50106	16.30712	85.04873	25.36178	17.98161	116.12908	57.71817	52.20388
0.3	87.46387	26.32307	22.16784	96.89897	30.35425	24.58655	150.59579	66.03779	61.29493
0.4	94.93003	31.1915	27.62545	107.28348	36.46305	30.75737	168.21587	74.37925	70.03639
0.5	101.78158	36.37002	33.11407	114.1932	40.77332	36.21498	179.03515	81.0533	77.39616
0.6	104.9637	39.62598	37.20727	118.47867	45.17662	40.61828	186.46528	85.92794	82.67079
0.7	106.60571	42.81992	40.92837	119.09353	49.14579	44.64946	191.35052	88.54245	85.62816
0.8	107.85272	46.69607	44.64946	119.06341	51.96762	48.1535	193.53765	89.15554	86.15554
0.9	107.95461	49.05276	47.40928	119.15732	55.00651	49.89001	193.02667	89.99811	86.99811
1.0	108.91137	52.49477	50.72725	119.19176	56.38633	53.53367	193.16441	90.56602	87.29811

## Data Availability

The original contributions presented in this study are included in the article, further inquiries can be directed to the corresponding authors.
